# A Combined Network Analysis for Orthorexia Nervosa, Obsessive Compulsive, and Eating Disorder Symptoms

**DOI:** 10.3390/nu18081179

**Published:** 2026-04-09

**Authors:** Caterina Lombardo, Silvia Cerolini, Andrea Zagaria, Mariacarolina Vacca, Rachel F. Rodgers

**Affiliations:** 1 Department of Psychology, Sapienza University of Rome, 00185 Rome, Italy; s.cerolini@unimarconi.it (S.C.); andrea.zagaria@uniroma2.it (A.Z.); mariacarolina.vacca@unicas.it (M.V.); r.rodgers@northeastern.edu (R.F.R.); 2 Department of Human Sciences, Università degli Studi Guglielmo Marconi, 00193 Rome, Italy; 3 Department of Systems Medicine, Tor Vergata University of Rome, 00133 Rome, Italy; 4 Department of Human, Social and Health Sciences, University of Cassino and Southern Lazio (UNICAS), 03043 Cassino, Italy; 5 Department of Experimental Medicine, Sapienza University of Rome, 00185 Rome, Italy; 6 Department of Applied Psychology (APPEAR Team), Northeastern University, Boston, MA 02115, USA; 7 Department of Psychiatric Emergency & Acute Care, Lapeyronie Hospital, CHRU Montpellier, 34090 Montpellier, France; 8 Unité de Recherche Clinique et d’Innovation, Hôpital du Cotentin, 50100 Cherbourg, France

**Keywords:** orthorexia nervosa, eating disorders, obsessive compulsive disorder, network analysis, bridge nodes, healthy eating

## Abstract

Orthorexia nervosa (ON) is a clinical condition characterized by rigid and inflexible rules about consuming healthy food, potentially leading to harmful consequences for physical and mental health and significant impairment in major life domains. Overlap or independence between ON and other clinical entities, like other eating disorders (EDs) or obsessive compulsive disorder (OCD), still needs clarification. **Objectives**: This study aims to examine the overlap versus independence of core symptoms of ON from these two classes of disorders using a network approach. **Methods**: A group of 422 university students (71.8% females), with a mean age of 20.70 years (SD = 3.44), completed questionnaires assessing those symptoms. **Results**: Results revealed that no ON symptoms were nested within the OCD and ED clusters, and vice versa, thereby supporting their empirical distinctiveness. Although the symptoms were organised into distinct communities, ON symptoms were more strongly linked to EDs than to OCD. Bridge symptom analyses revealed that the nodes “*Emotional consequences due to healthy eating*”, “*Worry about healthy food*”, and “*Obsessing*” exhibited the highest bridge strength connecting clusters of ON, EDs, and OCD symptoms. Conversely, the nodes “*Food safety*” and “*Ordering*” showed the lowest bridge strength, suggesting that these nodes played only a marginal role in linking ON, EDs, and OCD. **Conclusions**: From a nosological perspective, the findings provide empirical support for conceptualizing ON as a distinct yet symptomatically related condition within the broader spectrum of eating-related psychopathology, while acknowledging that definitive nosological classification requires further longitudinal and clinical research.

## 1. Introduction

Orthorexia nervosa (ON) is a clinical condition characterized by rigid and inflexible rules about consuming healthy food, potentially leading to harmful consequences for physical and mental health and significant impairment in major life domains [1,2,3]. It was first described by Steven Bratman in 1997 as a condition marked by pathological fixation on consuming healthy foods and rigid eating behaviors, which can negatively impact mental and physical health [4,5]. According to the findings of a recent consensus-building study [1], ON is characterized by a strong preoccupation with one’s eating behavior and self-imposed rigid rules, including spending excessive time planning, obtaining, preparing, and/or eating healthy food.

ON is also associated with reluctance to eat food prepared by others to avoid eating certain types of foods considered unhealthy, extreme preoccupation around eating only organic or “clean” foods, and excessive concern about food quality [1]. ON is not centered around weight and shape preoccupation or restrictive eating for weight loss, but rather on a pathological preoccupation with the perceived healthiness and purity of food. Although food quality is often invoked in descriptions of ON, it should not be equated with healthiness per se. In orthorexia, the central concern is not food quality or sustainability, but the moralized and health-driven evaluation of foods based on their presumed effects on physical well-being. Thus, restrictive behaviors in ON are primarily motivated by the pursuit of optimal health and the avoidance of perceived “unhealthy” foods, rather than by weight or shape concerns [1]. Nevertheless, this may still result in severe restrictions [6] that can lead to weight loss, nutritional deficiencies, and hormonal disturbances, and affect crucial psychosocial functioning areas [1,5]. Furthermore, these restrictive eating behaviors may be accompanied by onerous rituals related to food selection and preparation [7].

ON has been associated with multiple areas of impairment including emotional consequences (e.g., feelings of guilt or shame following the consumption of unhealthy food), cognitive consequences (e.g., struggles with maintaining focus and attention), and social consequences (e.g., social isolation), all of which can negatively impact an individual’s educational, occupational, and social functioning [1]. Despite this growing body of work, numerous aspects of ON continue to need additional clarification. Currently, ON is not yet recognized as a mental disorder in the DSM-5-TR [8] or other diagnostic systems, and it is still unclear whether it would be best conceptualized within the spectrum of eating disorders do [1] or as a variant of obsessive compulsive disorders [9] or as a separate diagnostic category of its own [10]. The consensus study [1] involving 47 experts on eating disorders and ON research and clinical practice, suggests that ON could be considered a mental health disorder within the Feeding and Eating Disorder (FED) spectrum, supporting previous meta-analytic evidence (e.g., [2]) demonstrating a moderate overlap between ON and FED symptoms, attributed to shared core features such as intense food concerns, rigid dietary rules, the strong link between diet and self-esteem, and the social and health consequences. However, ON seems to maintain its conceptual distinctiveness compared to other FEDs, with the main differences being: (a) its emphasis on food quality rather than quantity, (b) core preoccupations focusing on avoiding unhealthy foods and overestimating the effects of food quality on physical health, and (c) the absence of weight or shape concerns [1]. Overall, the consensus study also highlighted the presence of several similarities and shared symptoms with OCD: Both ON and OCD share cognitive rigidity, perfectionism traits, and obsessions. However, ON and OCD seem to differ in their focus on eating behavior and health: In ON, the obsessions and compulsions are mainly ego-syntonic and directed to healthy food, while OCD involves ego-dystonic obsessions and suppressing unwanted thoughts [1].

The literature reviewed above clarifies why evaluating the overlap and or independence among ON and other clinical entities is an important gap. Clarifying the status of ON within existing diagnostic frameworks represents an important challenge for contemporary psychopathology. To date, uncertainty remains regarding whether ON is a distinct clinical manifestation, a specific marker of OC spectrum, or an additional ED condition. The clarification of the nosographic status of ON may help support the development of more appropriate assessment and intervention strategies and treatments.

In recent years, network analysis has emerged as a novel and useful framework for the study of mental disorders. This framework argues that mental disorders can be conceptualized as networks of mutually reinforcing symptoms [11]; groups of symptoms cohere as a syndrome, not because they are caused by an underlying latent disorder, but because they are connected through a web of causal relationships [12]. The network approach has proven useful in the study of eating disorders more generally and has increased our understanding of their nature and characteristics (e.g., [12,13,14]). Importantly, network analysis has also been applied to investigations of the overlap between the networks of symptoms of disorders that share common features [15,16]. Thus, this approach seems particularly well-suited to further exploring the characteristics of ON in comparison with the other disorders with which it shares several characteristics, namely EDs and OCD.

Within this framework, this study aimed to examine the overlap versus independence of core symptoms of ON and those of OCD on one hand, and EDs on the other hand, using the network approach to better characterize the nature of ON and inform its classification. In this context, network analysis provides a useful tool for exploring whether ON represents a distinct cluster of symptoms or is embedded within the symptom configuration of EDs or OCD. Given the literature reviewed above, we hypothesized that network strength would be greater among ON and ED core symptoms as compared to ON and OCD core symptoms. Additionally, we expect to identify ON as a separate (though associated) clinical condition from both EDs and OCD.

## 2. Materials and Methods

### 2.1. Participants

The sample consisted of 422 university students (71.8% females, 28.2% males), with a mean age of 20.70 years (SD = 3.44; range = 18–53), enrolled at the Sapienza University of Rome. The mean Body Mass Index (BMI) was 21.83 kg/m^2^ (SD = 3.42). Of the total sample, 8.1% met the cut-off for clinically significant ON symptoms (DOS score ≥25 [17,18]). Additionally, 31.3% of the students scored above the cut-off for clinically significant ED symptoms on the DEQ (score ≥30 [19]). Finally, 14.9% of participants scored above the cut-off on the OCI-R (≥21 [20], suggesting clinically significant OCD symptoms. Regarding comorbidity rates, 6.7% of the sample met the cut-offs for both ON and ED symptoms, while 2.7% met the cut-offs for both ON and OCD symptoms. Additionally, 2.5% of participants met the cut-offs for ON, ED, and OCD symptoms simultaneously.

Students gave their written informed consent and volunteered to participate in the study. In large group sessions during their lecture time from September 2019 to January 2020, all participants completed self-report questionnaires, completion requiring approximately half an hour. As network analysis is still an emerging field, rules of thumb regarding sample size have not yet been established. However, previous studies examining the overlap of core symptoms from different diagnoses have included samples ranging from *n* = 196 to *n* = 408 [11]. Therefore, the current sample size seems to be more than adequate for the purposes of the present study. The study was approved by the Institutional Review Board of the Department of Psychology at Sapienza University of Rome (Prot. n. 0000227).

### 2.2. Measures

#### 2.2.1. Demographic Information

Demographic information (sex and gender, education level, socio-economic status) and information related to specific dietary restrictions (vegan, celiac disease, etc.) and the presence of cardio-metabolic diseases and prescription medication were collected.

#### 2.2.2. Orthorexia Nervosa Symptoms

The ORTO-15 [7] includes 15 items assessing orthorexia behaviors and attitudes divided across 3 subscales: cognitive aspects, clinical concerns, and emotional factors. A cut-off score of 40 was originally identified as reflecting the presence of ON (scores ranging from 15 to 60, efficacy 73.8%, sensitivity 55.6%, and specificity 75.8%). Although ORTO-15 has been criticized in the literature for its limited criterion validity and tendency to overestimate orthorexia prevalence when total scores and cut-offs are applied (e.g., [16]), it is important to clarify that in the present study, the instrument was not used for diagnostic or classificatory purposes. Specifically, we did not rely on total scores nor apply any cut-off criteria. Instead, only two selected individual items (item 6 and item 10) were included as observable indicators of specific orthorexic symptoms (see the following Section 2.3 for more details).

The Düsseldorf Orthorexia Scale [17], a 10-item scale developed in Germany to assess symptoms of Orthorexia Nervosa, was used in the Italian version [18]. Higher scores indicate more pronounced orthorexic behavior, with a maximum score of 40 points. The presence of ON is indicated by a cutoff score of ≥25. The scale demonstrated good psychometric properties (Cronbach’s alpha = 0.84 and test–retest reliability, r = 0.79) [17]. To identify the core ON symptomatology, the following items were selected: items 1, 2, 3, 4, 6, and 8. See the next Section 2.3 for more details.

#### 2.2.3. Eating Disorder Symptoms

The Disordered Eating Questionnaire [21] is a 24-item scale assessing eating disorder symptoms based on DSM criteria. It has a cut-off score of 30 and excellent psychometric properties [19,21] and a Cronbach’s alpha = 0.90.

The Eating Attitudes Test-26 (EAT-26, [22]) is a 26-item scale assessing preoccupation and symptoms related to eating disorders. The Italian version validated by Dotti and Lazzari (1998) [23] was used. It consists of 3 subscales: “dieting”, “bulimia and food preoccupation”, and “oral control”. Cronbach’s alpha ranges from 0.66 to 0.86.

#### 2.2.4. Obsessive Compulsive Disorder Symptoms

The Obsessive Compulsive Inventory-Revised [20], in the Italian version of Sica and colleagues (2009) [24], is an 18-item self-report scale assessing obsessive compulsive symptoms. It includes 6 subscales (Contamination, Obsessions, Hoarding, Ordering, Checking, and Neutralizing) with good to excellent psychometric properties. The subscale “Hoarding” was not considered for the purpose of this study since hoarding symptoms are currently not included within the OCD category, as it has been conceptualized as a distinct diagnostic entity in DSM-5-TR [8]. Cronbach’s alpha ranges from 0.69 to 0.85.

### 2.3. Node Selection

The nodes of this network analysis were selected referring to the most updated theoretical framework for Orthorexia Nervosa, Eating Disorders, and Obsessive Compulsive Disorder. Specifically, the choice of nodes representing ON symptoms was guided by the latest ON conceptualization on definition and diagnostic criteria by the consensus of experts in the field [1]. Specifically, the core symptoms of the core nodes of orthorexia symptomatology included in our network are derived from the Düsseldorf Orthorexia Scale (DOS), which is considered a psychometrically robust and conceptually grounded measure of ON. Specifically, the following nodes capture central features of orthorexia by items 1, 2, 3, 4, 6, and 8. These nodes reflect the core phenomenology of ON as described in recent consensus definitions [1], including cognitive preoccupation, rigid dietary rules, emotional distress, and social impairment. Only two items of ORTHO-15 (items 6 and 10) were used to represent the behavioral commitment and prioritization of healthy eating (i.e., economic investment in healthy eating) and the overvaluation of dietary behavior in self-concept (i.e., self-esteem linked to healthy eating), with both dimensions increasingly recognized in the recent conceptualization of ON by the consensus [1]. Secondly, the choice of the nodes for EDs, representing the core psychopathology, was based on the results of a systematic review by Monteleone and Cascino (2021) [25] of twenty-five studies employing the network approach for diagnosis, comorbidity, and treatment outcome for EDs. Lastly, the choice of OCD nodes refers to the main core psychopathology highlighted by a recent network analysis by Berle et al. (2023) [26] examining the network of OCI-R subscales. All of the selected items, contents, and referring scales are summarized in Supplementary Materials (Document S1).

Importantly, node selection was driven by theoretical coherence rather than by adherence to scale total scores. In line with previous network studies (e.g., [12,14,16]), individual items were selected to represent clinically meaningful symptom indicators derived from established conceptual frameworks. Thus, the focus of the present study was on modeling the interplay between specific behavioral and cognitive manifestations, rather than on validating or comparing psychometric instruments. Moreover, within a psychopathological network analytic framework, nodes represent directly interacting symptoms rather than reflective indicators of an underlying latent construct. Consequently, concerns related to internal consistency or dimensionality, which are central in latent variable modeling, are less directly applicable when the focus is on symptom-level associations. Despite the well-known psychometric limitations of the ORTO (e.g., low internal consistency) [16], the selected ORTO items were chosen because they map onto core features identified in recent consensus-based conceptualizations of orthorexia nervosa [1], including the economic impact of healthy eating or the self-esteem associated with healthy eating, which were not represented in the DOS.

### 2.4. Data Analytic Strategy

We conducted a psychometric network analysis in R (version 4.4.3) using the bootnet [27], qgraph [28], glasso [29], and networktools [30] packages. The symptom network was estimated via a Gaussian Graphical Model (GGM) by applying the graphical least absolute shrinkage and selection operator regularization (gLASSO, [31]) in combination with the Extended Bayesian Information Criterion model selection (EBIC [32]; hyperparameter γ = 0.50). This regularization approach yields an interpretable, sparse network by shrinking spurious partial correlations to exactly zero, thereby retaining only the most important empirical relationships in the data [27]. Within the GGM, nodes represent observed variables (i.e., symptoms) linked by undirected edges. Edges correspond to partial correlation coefficients, indicating the strength of the conditional associations between pairs of nodes after controlling for all other variables in the network. The network layout was based on the Fruchterman–Reingold algorithm [33], with positive edges shown in blue, negative edges in red, and edge thickness corresponding to the magnitude of the associations.

To examine the potential elements accounting for the interrelatedness of these symptoms, bridge centrality indices were computed, including bridge strength and bridge expected influence [34]. Bridge strength is defined as the sum of the absolute values of all edge weights between a given symptom *X* and all symptoms belonging to other clusters. Similarly, bridge expected influence refers to the sum (positive or negative) of all edge weights linking a given symptom *X* with symptoms in different clusters. These metrics are useful to identify bridge symptoms, defined as nodes connecting distinct symptom clusters (e.g., ON with OCD), which may play a central role in the development and maintenance of comorbid mental disorders [34].

Finally, network accuracy and stability were assessed using a two-step bootstrapping procedure [27]. First, edge weight accuracy was evaluated by computing non-parametric bootstrap confidence intervals based on 1000 resamples, with narrower intervals indicating more precise estimates. Second, the stability of centrality indices was assessed using case-dropping subset bootstrapping (1000 replications), from which the correlation stability (CS) coefficient was derived. The CS coefficient quantifies the maximum proportion of cases that can be dropped to retain, with 95% probability, a correlation ≥0.7 between statistics derived from the original network and those obtained from the trimmed samples. Following established guidelines [27], a CS coefficient exceeding 0.25 and 0.50 indicated acceptable and strong stability of the centrality estimates, respectively.

## 3. Results

### 3.1. Descriptive Statistics

Descriptive statistics for the nodes are reported in Table 1. Significant deviations from univariate normality were observed (skewness and kurtosis > |2| [35]). Therefore, to relax the GGM assumption of normality, a non-paranormal transformation (i.e., Gaussianization) was applied to the data prior to further analyses (e.g., [36]).

### 3.2. Psychometric Network Analysis

**Figure 1:**
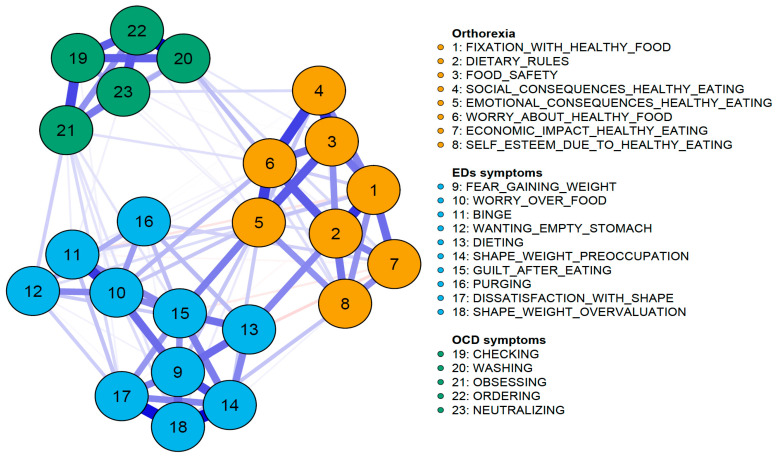
EBICglasso network. Notes: Blue lines represent positive edges, while red lines represent negative edges. The thickness of the edges corresponds to their weights. ON symptoms are depicted in orange, ED symptoms in sky blue, and OCD symptoms in green.

Figure 1 illustrates the EBICglasso network, which includes eight ON nodes, ten EDs nodes, and five OCD nodes. The network was quite sparse, with 111 out of the 253 possible edges (43.9%) estimated to be higher than zero. As shown in Figure 1, ON nodes are clustered in the right-central area of the network, OCD symptoms are grouped in the upper-left region, and ED symptoms are located in the lower portion. Despite the substantial interrelations between these clusters of symptoms, no ON symptoms were nested within the OCD and ED clusters, and vice versa, thereby supporting their empirical distinctiveness.

Although the symptoms were organized into distinct communities, analyses of inter-cluster connectivity revealed that ON symptoms were more strongly linked to ED symptoms than to OCD symptoms. Specifically, ON–ED associations exceeded ON–OCD both in number (23 vs. 12 non-zero edges) and in average strength (mean absolute partial correlation = 0.044 vs. 0.030), suggesting that ON, while empirically distinct, was more closely interconnected with EDs than with OCD symptoms.

**Figure 2:**
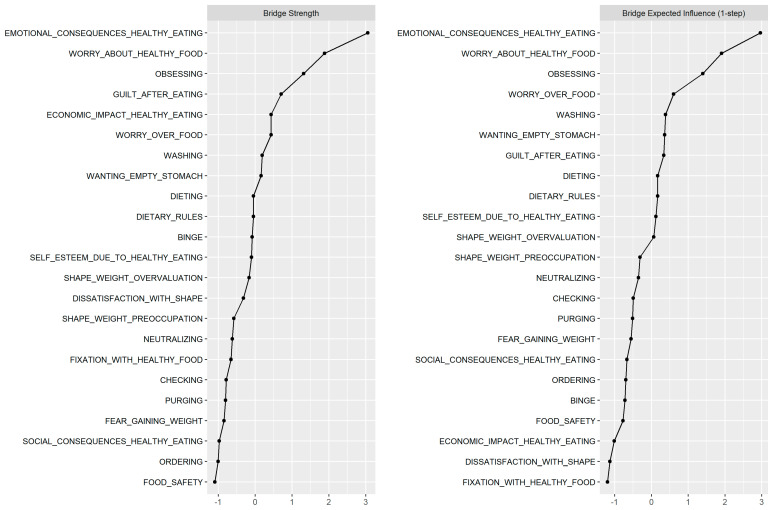
Bridge strength estimates. Estimates were z-scored (with M = 0 and SD = 1) to aid interpretation.

Furthermore, bridge symptom analyses revealed that the nodes “Emotional consequences due to healthy eating”, “Worry about healthy food”, and “Obsessing” exhibited the highest bridge strength and expected influence values (>1; see Figure 2). These estimates suggest that these three nodes had multiple and/or particularly strong inter-cluster connections, thus bridging the theoretically defined clusters of ON, EDs, and OCD symptoms. Conversely, the nodes “Food safety” and “Ordering” exhibited the lowest bridge strength (values <−1; see Figure 2), suggesting that these nodes played only a marginal role in linking ON, ED, and OCD symptoms.

The edge weight matrix, reporting exact partial correlations between nodes, is provided in Supplementary Materials (Document S2).

### 3.3. Stability and Accuracy of the Network

**Figure 3:**
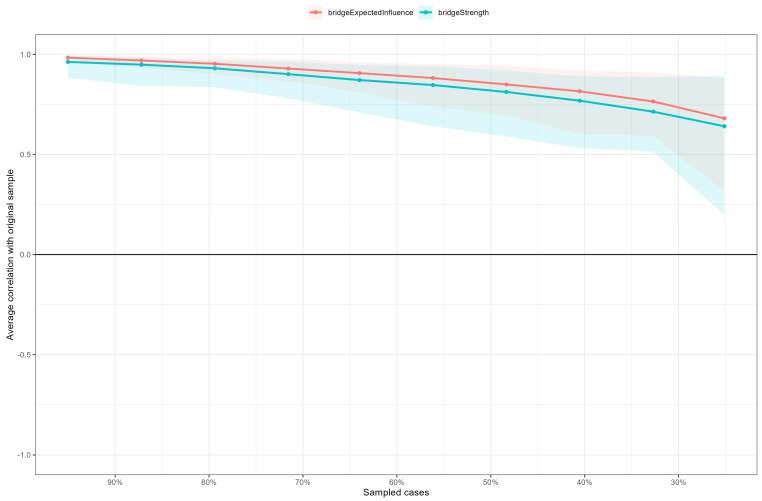
Stability of the bridge centrality metrics via case-dropping subset bootstrap. The x-axis reports the percentage of cases at each step. The y-axis reports the average correlations between centrality indices of networks with the original sample and those re-estimated with an increasingly higher percentage of dropped-out cases.

With respect to the stability of the centrality measures, the CS coefficient for bridge expected influence and strength, estimated via the case-dropping subset bootstrap, was 0.52 and 0.44, respectively (see Figure 3). Both values exceed the recommended threshold of 0.25, supporting the robustness and interpretability of the centrality metrics (e.g., [27,37]). Regarding accuracy, non-parametric bootstrapped confidence intervals indicated that the network model was reliably estimated, as shown by the relatively narrow intervals around the estimated edge weights (see Supplementary Document S2, Figure 1).

## 4. Discussion

The present study employed an EBICglasso network analysis to examine the relationships among ON, ED, and OCD symptoms. The findings contribute to the ongoing debate regarding the classification of ON within the psychopathological spectrum by elucidating its connectivity with EDs and OCD.

First, several correlations between symptoms were identified, providing insights into the core interactions within ON, EDs, and OCD. More specifically, results showed small but significant relationships between ON—“Dietary rules” and EDs—“Dieting”, and ON—“Emotional consequences due to healthy eating” and EDs—“Guilty after eating”. The first association supports the common pathway of cognitive rigidity in both ON and EDs, with individuals adhering to strict eating patterns and thus limiting their food intake. However, rigid dietary rules in ON focus on food quality and purity, whereas in EDs they are primarily driven by a desire for weight loss and body shape concerns [6,38]. Eating restriction is a central symptom in EDs. As highlighted by Fairburn (2008) [39], restriction and the total avoidance of certain foods is present in almost all eating disorders, although only in anorexia nervosa, restricting type, is it effective in terms of achieving low weight. In addition, individuals with ON often eliminate highly processed foods to avoid pesticides or artificial ingredients and prevent chronic conditions or enhance overall health, thus engaging in a sometimes highly restrictive and rigid diet without guidance from a medical professional [40]. Typically, this results in a strict reduction of food intake and the exclusion of certain food groups, leading to significant malnutrition and excessive weight loss [41]. Research highlights that dietary restraint, regardless of its motivation (e.g., weight/shape concerns, self-regulatory control; [42,43]) is associated with increased psychological distress and maladaptive eating behaviors [5,6]. Additionally, excessive cognitive control over eating in ON may increase vulnerability to ED behaviors, as the rigid rules may yield more generalized forms of restrictive eating, blurring the boundaries between these conditions [1]. Thus, while the underlying motivations may differ, the mechanisms of cognitive rigidity and eating restriction and their psychological consequences exhibit notable similarities, explaining the strong associations seen in our data.

A second result that should be outlined here is the association found between ON— “Emotional consequences due to healthy eating” and EDs—“Guilty after eating”. This result may highlight a second shared underlying psychological mechanism between ON and EDs. In both conditions, the imposition of strict dietary rules leads to emotional distress when these rules are violated. In orthorexia, negative emotions arise when eating deviates from perceived health standards [7,44], while in EDs, guilt emerges from a loss of control or consumption of “forbidden” foods [45]. These shared patterns suggest that both disorders involve maladaptive coping strategies, where food becomes a means of managing anxiety and achieving a sense of control, ultimately exacerbating emotional distress [44].

Despite the substantial interrelations between these symptom clusters, no ON symptoms were embedded within the ED and OCD clusters, nor vice versa, further reinforcing ON’s conceptual distinction. These findings are consistent with those of prior studies that indicate that although ON shares cognitive and behavioral characteristics with EDs and OCD, its distinct emphasis on food quality—rather than concerns about weight/shape or intrusive distressing thoughts—sets it apart [1,5,12].

Three symptoms emerged as key bridge nodes connecting ON, EDs, and OCD, namely ON—“Emotional consequences due to healthy eating”, ON—“Worry about healthy food” and OCD—“Obsessing”. These symptoms exhibited high bridge strength centrality (values >1 ), suggesting that they may act as potential mechanisms facilitating symptom interaction across disorders. First, emotional consequences due to healthy eating involve intense distress when individuals perceive that their food choices do not align with their rigid standards for health. This emotional distress is a hallmark of both EDs and OCD, where maladaptive perfectionistic tendencies often lead to significant psychological discomfort [46]. In ON, the focus on “healthy” eating creates an excessive concern with food quality, which in turn contributes to negative emotions when these standards are violated, a pattern resembling the distress associated with eating and weight-related concerns in EDs [47]. Similarly, in OCD, intrusive thoughts and compulsive behaviors related to food rituals can trigger emotional reactivity, reflecting the shared cognitive and emotional mechanisms between ON and OCD [48].

Second, worry about healthy food in ON, characterized by excessive preoccupation with the purity and nutritional value of food, closely aligns with the rigid, intrusive thoughts seen in OCD [49]. This obsessive focus on health could also act as a bridge to EDs, where worry about body image and food intake leads to disordered eating behaviors. The constant vigilance over food choices can trigger anxiety, a common feature in both ON and OCD, and contributes to the development of unhealthy eating patterns, as seen in EDs [50].

Third, “Obsessing” may serve as a critical link between ON and OCD, highlighting the role of rigid thought patterns and compulsive concerns about food in ON [2,38]. Research has demonstrated that both ON and OCD share cognitive and neurobiological features, particularly in terms of high levels of cognitive preoccupation and the persistence of intrusive thoughts [51]. In ON, obsessive focus on food often leads to compulsive behaviors, such as rigid eating habits and avoidance of certain foods, which are employed to reduce the anxiety caused by obsessive thoughts about health [52]. Similarly, in EDs, individuals may obsess over their weight, food intake, or body image, and engage in compensatory behaviors, such as restrictive eating or excessive exercise, to mitigate distress [9].

Conversely, ON—“Food safety” and OCD—“Ordering” had the lowest bridge strength (values < −1), indicating that these symptoms played a marginal role in inter-cluster connectivity. This finding suggests that these two nodes may not serve as a primary link [15] connecting ON and OCD domains. Specifically, the food safety concern in ON seems to differ qualitatively from the contamination obsessions in OCD (as indicated by the OCD—”Washing” node) despite the apparently similar nature of the content related to those domains.

Identifying bridge nodes in network analysis between ON, ED, and OCD symptoms provides important insights. Bridge nodes, defined as variables or symptoms that connect distinct clusters in a network, serve as links that facilitate interactions between different symptom groups [53]. Understanding these interconnections has implications for clinical practice, suggesting that targeted interventions that address shared mechanisms may be more effective in treating these overlapping conditions. 

The stability of the centrality network metrics was supported by CS-coefficients exceeding the recommended threshold of 0.25 [27]. Furthermore, bootstrap confidence intervals for edge weights were relatively narrow, indicating that the estimated relationships between symptoms were statistically reliable [11]. These results strengthen the validity of the observed network structure and provide a solid empirical basis for future research on ON’s classification.

While the study provides valuable insights, it is important to acknowledge several limitations. The cross-sectional design captures associations rather than causal relationships, and future longitudinal research will be necessary to explore causal pathways between ON, ED, and OCD symptoms [54]. Additionally, the study’s focus on a non-clinical sample of young adults limits the generalizability of the findings to clinical populations with severe ON or comorbid conditions [11]. Furthermore, the reliance on self-report questionnaires introduces the potential for response biases, highlighting the need for future studies to incorporate clinical interviews and behavioral assessments to improve diagnostic accuracy [20]. An additional methodological concern results from the inclusion of selected items from the ORTO-15. Although this instrument has been widely debated due to psychometric limitations when used as a total score measure, in the present study, it was employed exclusively at the item level to index specific orthorexic behaviors and cognitions consistent with current consensus criteria [1] that are not represented in the Düsseldorf Orthorexia Scale (i.e., economic impact of healthy eating and self-esteem associated with healthy eating), precluding a direct one-to-one substitution. Although we acknowledge that the ORTO-15 as a whole has limitations, we argue that the specific items selected in our study capture theoretically meaningful constructs, and, crucially, are not used in isolation but embedded within a network whose core structure is defined by DOS-derived nodes. Importantly, our analytical approach further mitigates concerns regarding the psychometric limitations of the full questionnaire. Network analysis operates at the level of observed variables (items) and focuses on the pattern of relationships between them, rather than on latent constructs, diagnostic thresholds, or total scores. As such, it is less dependent on the overall validity of a scale as a diagnostic instrument and instead allows for the examination of how specific behaviors and cognitions interact.

This approach has been widely adopted in the field, including in studies using instruments that have been subject to psychometric debate. For example, network analyses have been conducted using the Eating Disorder Examination Questionnaire (EDE-Q) [55], despite well-documented concerns regarding its factor structure and measurement properties. These studies show that network models can yield meaningful insights even when based on imperfect measures, provided that the included items are theoretically interpretable. However, future studies may benefit from developing consensus-aligned item pools specifically designed for network modeling of orthorexia symptoms.

Finally, data collection for the present study was conducted immediately before the onset of the COVID-19 pandemic. Previous research indicates an association between the pandemic and ED psychopathology overall. Data collection was conducted between September 2019 and January 2020, immediately prior to the global spread of COVID-19. Although the pandemic has been associated with changes in eating-related psychopathology [54] as well as an exacerbation of ON symptoms across multiple international cohorts [55], the present findings reflect symptom associations observed in a pre-pandemic context. While mean symptom levels may fluctuate across historical periods, the structural configuration of symptom networks may demonstrate greater temporal stability. Nonetheless, replication in post-pandemic samples is warranted to examine the robustness of the observed inter-symptom associations across different sociocultural contexts. Future research should also aim to replicate these findings in samples that are more demographically balanced and representative of the general population, particularly in terms of gender and age distribution, to further enhance the external validity of the study.

## 5. Conclusions

The present study provides a novel contribution by examining the network structure of ON in relation to EDs and OCD. However, the interpretation of the network structure and its nosological implications is based on the overall pattern of associations observed across ON, ED, and OCD symptoms, rather than on single nodes. Alternative operationalizations of ON and/or different measurement approaches are warranted to further refine the understanding of these clinical entities. Despite the limitations highlighted, our findings provide insights into the positioning of ON within the broader psychopathological landscape, particularly in terms of its symptom connectivity and potential classification as a distinct disorder. Future research using longitudinal methods should aim to clarify further the transdiagnostic models of eating pathology that include ON symptoms. Beyond methodological contributions, the present findings also have broader theoretical and clinical implications. From a nosological perspective, the findings shed new light on research examining the transdiagnostic mechanisms underlying ON. These intrinsic processes may connect differential clusters of symptoms, explaining the clinical co-occurrence between ON, OCD, and EDs. More specifically, emotional consequences due to healthy eating, worry about healthy food, and obsessing may facilitate symptom interactions and clinical comorbidity across these domains. Furthermore, from a clinical perspective, identifying bridge symptoms may also inform intervention strategies. Indeed, according to the network theory of psychopathology [15], bridges could represent a potential clinical target of transdiagnostic interventions. Future studies are warranted to further evaluate the effectiveness of therapeutic approaches addressing these mechanisms.

## Figures and Tables

**Table 1:** Descriptive statistics of the nodes under investigation. Abbreviations: SD, standard deviation.

Node	Mean	SD	Skewness	Kurtosis
Fixation with healthy food	1.90	0.83	0.59	−0.36
Dietary rules	2.16	0.97	0.20	−0.97
Food safety	1.40	0.63	1.33	1.34
Social consequences due to healthy eating	1.22	0.55	2.54	7.11
Emotional consequences due to healthy eating	1.68	0.89	1.08	0.34
Worry about healthy food	1.39	0.73	1.87	2.99
Economic impact due to healthy eating	2.64	0.80	−0.04	−0.49
Self-esteem related to healthy eating	2.10	0.97	0.42	−0.86
Fear of gaining weight	0.65	1.02	1.25	0.11
Worry over food	0.23	0.67	2.97	7.96
Binge	0.23	0.65	3.00	8.53
Wanting empty stomach	0.13	0.52	4.41	19.52
Dieting	1.36	1.48	0.96	−0.04
Shape weight preoccupation	1.87	1.70	0.58	−1.01
Guilt after eating	1.07	1.38	1.19	0.41
Purging	0.04	0.25	7.29	62.77
Dissatisfaction with shape	2.02	1.99	0.58	−1.02
Shape and weight overvaluation	2.34	2.08	0.34	−1.27
Checking	0.81	1.01	1.19	0.74
Washing	0.64	0.98	1.66	2.28
Obsessing	0.84	1.11	1.17	0.44
Ordering	0.57	0.95	1.73	2.28
Neutralizing	0.28	0.78	3.03	8.95

## Data Availability

The data that support the findings of this study are available from the corresponding author upon reasonable request.

## Supplementary Materials

The following supporting information can be downloaded at https://www.mdpi.com/article/10.3390/nu18081179/s1: Supplementary Document S1: NODES; Supplementary Document S2: RESULTS.

## Abbreviations

The following abbreviations are used in this manuscript:

ON	Orthorexia Nervosa
OCD	Obsessive Compulsive Disorder
EDs	Eating Disorders
GGM	Gaussian Graphical Model
