# Supplementary material for: A Combined Network Analysis for Orthorexia Nervosa, Obsessive Compulsive, and Eating Disorder Symptoms
Source: Nutrients. 2026 Apr 9;18(8):1179. doi: 10.3390/nu18081179 (PMC13119138; doi:10.3390/nu18081179)
Supplement: Supplementary file 1 [file nutrients-18-01179-s001.zip › Supplementary Document S1_NODES_rev.pdf]

ORTHOREXIA NERVOSA			
**Node**	**Scale**	**Item n.**	**Item content**
1. FIXATION WITH HEALTHY FOOD	DOS	1	*"Per me, mangiare cibi sani è più importante del piacere di mangiare"*
2.DIETARY RULES	DOS	2	*"Ho stabilito delle regole per la mia alimentazione"*
3.FOOD SAFETY	DOS	3	*"Posso gustare un cibo solo se sono certa/o che sia sano"*
4.SOCIAL CONSEQUENCES DUE TO HEALTHY EATING	DOS	4	*"Cerco di evitare inviti da amici che non siano attenti a una sana alimentazione"*
5.EMOTIONAL CONSEQUENCES DUE TO UNHEALTHY EATING	DOS	6	*"Se mangio qualcosa di non sano, mi sento molto in colpa"*
6.WORRY ABOUT HEALTHY FOOD	DOS	8	"I miei pensieri girano sempre intorno a un'alimentazione sana e regolo di conseguenza il corso della mia giornata"
7.ECONOMIC IMPACT OF HEALTHY EATING	ORTHO-15	6	*"E' disposto a spendere di più per avere un cibo sano?"*
8.SELF-ESTEEM DUE TO HEALTHY EATING	ORTHO-15	10	*"Ritiene che la convinzione di alimentarsi con cibi sani aumenti la sua autostima?"*
EATING DISORDERS			
**Node**	**Scale**	**Item n.**	**Item content**
9.FEAR OF GAINING WEIGHT	EAT-26	1	*"Ho una terribile paura di ingrassare"*
10.WORRY OVER FOOD	EAT-26	3	*"Penso al cibo con preoccupazione"*
11.BINGE	EAT-26	4	*"Mi è capitato di mangiare con enorme voracità sentendomi incapace di smettere"*
12. WANTING AN EMPTY STOMACH	EAT-26	24	*"Mi piace avere lo stomaco vuoto"*

13.DIETING	DEQ	A1	*“Limitare la quantita’ di cibo o calorie assunte, per ridurre il tuo peso”*
14. SHAPE AND WEIGHT PREOCCUPATION	DEQ	A6	*“Trascorrere molto tempo pensando al tuo peso o all’aspetto di alcune parti del tuo corpo “*
15. GUILT AFTER EATING	DEQ	A9	*“Sentirti in colpa dopo aver mangiato”*
16. PURGING	DEQ	A13	*“Provocarti volutamente il vomito per controllare il tuo peso”*
17. DISSATISFACTION WITH SHAPE	DEQ	B2	*“Ti ha creato disagio vedere il tuo corpo riflesso in uno specchio”*
18.SHAPE AND WEIGHT OVERVALUATION	DEQ	B6	*“La stima che hai di te stessa/o e’ stata influenzata (in positivo o in negativo) dal pensiero del tuo peso o dell’aspetto di alcune parti del tuo corpo”*
OBSESSIVE COMPULSIVE DISORDER			
**Node**	**Scale**	**Item n.**	**Item content**
19.CHECKING	OCI-R	2	*“Ho la tendenza a controllare e ricontrollare le cose molto più spesso del necessario”*
20.WASHING	OCI-R	11	*“Qualche volta devo lavarmi o pulirmi semplicemente perché mi sento contaminato”*
21.OBSESSING	OCI-R	12	*“Sono turbato da pensieri spiacevoli che entrano nella mia mente contro la mia volontà”*
22.ORDERING	OCI-R	15	*“Ho bisogno che le cose attorno a me siano sistemate secondo un particolare ordine”*

23.NEUTRALIZING	OCI-R	16	*“Ho la sensazione che ci siano numeri buoni e numeri cattivi”*
