# Supplementary material for: A Combined Network Analysis for Orthorexia Nervosa, Obsessive Compulsive, and Eating Disorder Symptoms
Source: Nutrients. 2026 Apr 9;18(8):1179. doi: 10.3390/nu18081179 (PMC13119138; doi:10.3390/nu18081179)
Supplement: Supplementary file 1 [file nutrients-18-01179-s001.zip › Supplementary Document S2_RESULTS_rev.pdf]

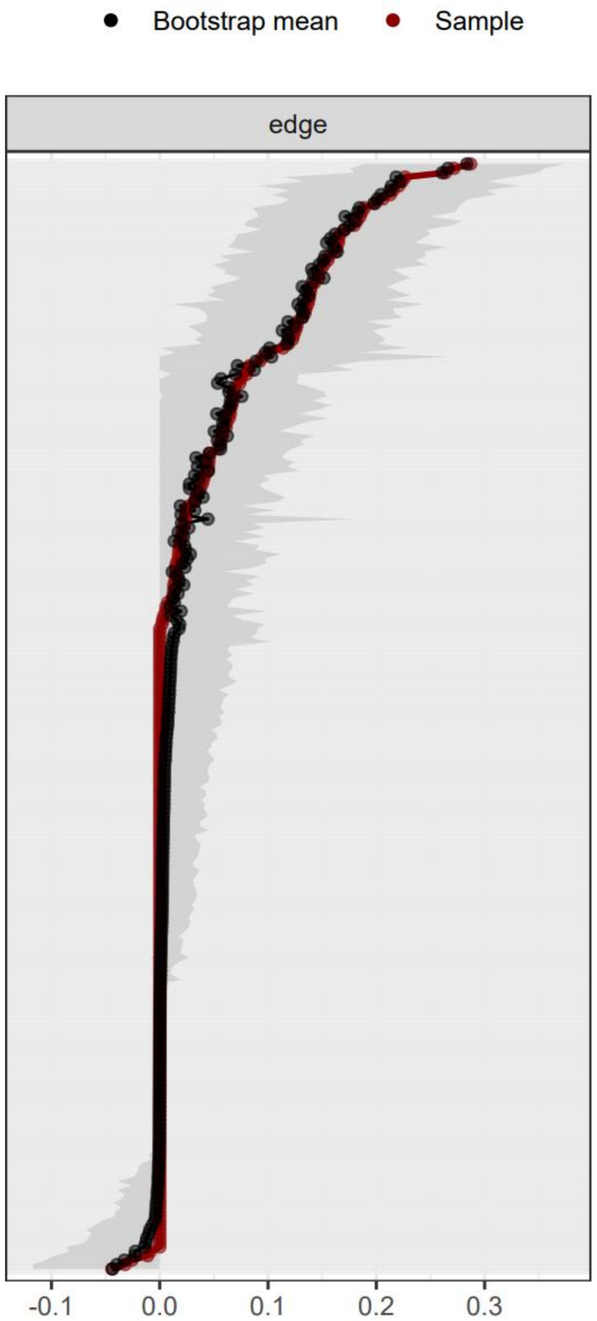

Supplementary Figure 1. The nonparametric bootstrapped confidence intervals of edge weights. The red dots indicate the sample values, the black dots indicate the bootstrap means, and the grey area indicates the 95% bootstrapped CIs. The wider the grey area, the lower the accuracy. Edge labels were omitted to preserve visual clarity and interpretability, as their inclusion would have substantially compromised the readability of the figure.

		ON NODES								EDS NODES										OCD NODES				
	Node	1	2	3	4	5	6	7	8	9	10	11	12	13	14	15	16	17	18	19	20	21	22	23
ON NODES	1	0	0.22	0.16	0.14	0.07	0.06	0.16	0.12	0	0	-0.03	0.04	0	0	0	0	-0.01	0	0	0	0	0	0
	2	0.22	0	0.13	0.01	0.05	0.18	0.14	0.15	0	0	0	0	0.14	0	0	0	0	0	0	0	0	0	0
	3	0.16	0.13	0	0.23	0.19	0.16	0	0.02	0	0	0	0.01	0	0	0	0	0	0	0	0	0	0.03	0.01
	4	0.14	0.01	0.23	0	0.07	0.21	0	0.02	0	0	0	0.01	0	0	0	0.01	0	0	0	0	0	0	0.05
	5	0.07	0.05	0.19	0.07	0	0.22	0	0.13	0.07	0.07	0.05	0	0	0.01	0.15	0	0	0.02	0.02	0	0	0.02	0.01
	6	0.06	0.18	0.16	0.21	0.22	0	0.02	0.04	0	0.08	0	0.02	0	0.06	0	0.01	0	0	0.02	0.07	0.04	0	0
	7	0.16	0.14	0	0	0	0.02	0	0.13	0	0	-0.01	0	0	0	-0.03	0.05	-0.04	0	0	0.04	0	0	0
	8	0.12	0.15	0.02	0.02	0.13	0.04	0.13	0	0	0	0	0	0	0.01	0	0	0	0.07	0	0.04	0	0	0.01
EDS NODES	9	0	0	0	0	0.07	0	0	0	0	0.17	0.02	0.01	0.17	0.2	0.08	0	0.15	0.17	0	0	0	0	0
	10	0	0	0	0	0.07	0.08	0	0	0.17	0	0.22	0.14	0.07	0	0.13	0.11	0	0.04	0	0	0.03	0	0
	11	-0.03	0	0	0	0.05	0	-0.01	0	0.02	0.22	0	0	0	0	0.13	0.02	0.07	0	0	0	0.02	0	0.02
	12	0.04	0	0.01	0.01	0	0.02	0	0	0.01	0.14	0	0	0.06	0	0	0.1	0.06	0.01	0.02	0	0.06	0	0
	13	0	0.14	0	0	0	0	0	0	0.17	0.07	0	0.06	0	0.14	0.16	0.08	0	0	0	0	0	0	0
	14	0	0	0	0	0.01	0.06	0	0.01	0.2	0	0	0	0.14	0	0.15	0	0.14	0.27	0	0	0	0	0
	15	0	0	0	0	0.15	0	-0.03	0	0.08	0.13	0.13	0	0.16	0.15	0	0.07	0.12	0.12	0	0	0.03	0	0
	16	0	0	0	0.01	0	0.01	0.05	0	0	0.11	0.02	0.1	0.08	0	0.07	0	0	0.01	0	0	0	0	0

		ON NODES								EDS NODES										OCD NODES				
	Node	1	2	3	4	5	6	7	8	9	10	11	12	13	14	15	16	17	18	19	20	21	22	23
	17	-0.01	0	0	0	0	0	-0.04	0	0.15	0	0.07	0.06	0	0.14	0.12	0	0	0.26	0.01	0	0.05	0	0
	18	0	0	0	0	0.02	0	0	0.07	0.17	0.04	0	0.01	0	0.27	0.12	0.01	0.26	0	0	0	0.03	0	0
OCD NODES	19	0	0	0	0	0.02	0.02	0	0	0	0	0	0.02	0	0	0	0	0.01	0	0	0.18	0.21	0.18	0.1
	20	0	0	0	0	0	0.07	0.04	0.04	0	0	0	0	0	0	0	0	0	0	0.18	0	0.09	0.29	0.06
	21	0	0	0	0	0	0.04	0	0	0	0.03	0.02	0.06	0	0	0.03	0	0.05	0.03	0.21	0.09	0	0.13	0.15
	22	0	0	0.03	0	0.02	0	0	0	0	0	0	0	0	0	0	0	0	0	0.18	0.29	0.13	0	0.19
	23	0	0	0.01	0.05	0.01	0	0	0.01	0	0	0.02	0	0	0	0	0	0	0	0.1	0.06	0.15	0.19	0

Supplementary Table 1. Edge weights matrix. All values are rounded to two decimal places.

Labels: 1 = Fixation with healthy food; 2 = Dietary rules; 3 = Food safety; 4 = Social consequences due to healthy eating; 5 = Emotional consequences due to healthy eating; 6 = worry about healthy food; 7 = Economic impact due to healthy eating; 8 = Self-esteem and healthy eating; 9 = Fear of gaining weight; 10 = Worry over food; 11 = Binge; 12 = Wanting empty stomach; 13 = Dieting; 14 = Shape and weight preoccupation; 15 = Guilt after eating; 16 = Purging; 17 = Dissatisfaction with shape; 18 = Shape and weight overvaluation; 19 = Checking; 20 = Washing; 21 = Obsessing; 22 = Ordering; 23 = Neutralizing.
